# General Practitioners’ Perceptions of Whether Teleconsultations Reduce the Number of Face-to-face Visits in the Catalan Public Primary Care System: Retrospective Cross-Sectional Study

**DOI:** 10.2196/14478

**Published:** 2020-03-16

**Authors:** Francesc López Seguí, Josep Vidal-Alaball, Marta Sagarra Castro, Anna García-Altés, Francesc García Cuyàs

**Affiliations:** 1 TIC Salut Social Ministry of Health Barcelona Spain; 2 Centre for Research in Health and Economics Pompeu Fabra University Barcelona Spain; 3 Health Promotion in Rural Areas Research Group Gerència Territorial de la Catalunya Central Institut Català de la Salut Sant Fruitós de Bages Spain; 4 Unitat de Suport a la Recerca de la Catalunya Central Fundació Institut Universitari per a la recerca a l'Atenció Primària de Salut Jordi Gol i Gurina Sant Fruitós de Bages Spain; 5 Centre d’Atenció Primària Capellades Gerència Territorial de la Catalunya Central Institut Català de la Salut Barcelona Spain; 6 Agency for Healthcare Quality and Evaluation of Catalonia (AQuAS) Catalan Ministry of Health Barcelona Spain; 7 Sant Joan de Déu Hospital Catalan Ministry of Health Barcelona Spain

**Keywords:** telemedicine, remote consultation, primary care, general practitioners

## Abstract

**Background:**

eConsulta is a teleconsultation service involving general practitioners (GPs) and patients. It is part of the information system belonging to Catalonia’s primary care service. It has been in operation since the end of 2015 in conjunction with face-to-face consultations with Primary Care Teams as one of the services offered in the patient’s Personal Health Folder.

**Objective:**

This study aimed to assess the ability of using eConsulta to reduce the number of face-to-face visits to Primary Care Teams.

**Methods:**

Using 13 categories proposed by the researchers, 18 GPs from the Central Catalonia Health Region retrospectively classified 2268 cases managed with eConsulta and indicated whether, in their opinion, the teleconsultations reduced the number of face-to-face visits.

**Results:**

There was broad consensus among the GPs that eConsulta has the potential to resolve patient queries for every type of consultation. eConsulta avoided the need for a face-to-face visit in 87.9% of cases. In addition, the GPs reported that the ease of access increased the demand for health care support in 27.7% of cases; otherwise, the patient would not have initiated the queries. Therefore, based on the equation (88% x [1-28%]), eConsulta could replace 63%-88% of conventional appointments. The most frequent uses of the teleconsultation service were for management of test results (35.2%), medical enquiries (16.0%), and the management of repeat prescriptions (12.2%). On average, the teleconsultations consisted of a mean 1.57 messages (SD 0.54 messages); 45.9% (1040/2268) of the teleconsultations consisted of 1 message, and the majority of the remaining teleconsultations consisted of 2-5 interactions. The patient initiated 60.0% (1361/2268) of the teleconsultations.

**Conclusions:**

Based on the GPs’ perceptions, eConsulta could replace 63%-88% of conventional appointments. Therefore, asynchronous teleconsultations between practitioners and patients in primary care could avoid interactions that have limited added clinical value.

## Introduction

### Teleconsultation in the Context of the Public Health System in Catalonia

The Catalan health system provides publicly financed universal health coverage that is free at the point of access, thus ensuring that everyone who lives in Catalonia has the right to health care. It is a mixed health model funded through taxes, with equal access to a wide range of benefits offered by a single publicly available network of health resources, not all of which are publicly owned. The network includes a range of organizations (eg, mutual societies, foundations, health consortiums) that have historically provided health care. One in four individuals in Catalonia has additional private health insurance, which does not forfeit their right to public health care [1].

Catalonia is a pioneer in the use of Information and Communication Technologies (ICT) in public health care. Over the last decade, the adoption of teleconsultations has overtaken the use of other forms of telemedicine [2]. Of the many existing projects, eConsulta is particularly noteworthy. It is an asynchronous teleconsulting service involving general practitioners (GPs) and members of the public connected to the electronic medical history systems of public primary health care. eConsulta was designed to complement face-to-face contact with Primary Care Teams (PCT) in Catalonia. The service was introduced in 2015 and was phased in gradually until 2017, when it became established as a service available to all PCTs. At present, over 90% of the teams have used the tool [3]. Despite the uptake at the institutional level, its use in everyday medical practice is still growing. Interactions via eConsulta are intended to replace low value-added GP visits such as the collection of laboratory results, issuing of repeat prescriptions, and routine follow-up consultations for existing patients who, due to improvements in their symptoms or positive test results, do not require the GP to conduct a physical examination.

From the patient’s point of view, eConsulta is one of the services offered in their Personal Health Folder, a personal digital space that provides access to personal health information, allowing them to make enquiries and perform specific actions. Following a secure authentication process, patients can access an interface that allows them to submit their query and attach files, if necessary. The portal can be customized and keeps a record of previous teleconsultations.

### Comparison of Teleconsultation and Face-to-face Consultations

A comprehensive study comparing the use of eConsulta and face-to-face visits has not yet been conducted in Catalonia. A survey carried out during the initial stages of the intervention showed that 70% of GPs saw it as “a tool which had the potential to reduce the number of face-to-face visits” [4]. Moreover, no conclusive relationship between the use of teleconsultations and a reduction in the frequency of face-to-face visits has been identified in studies published internationally [5]. What is clear is that the uptake of teleconsultation is still very low and needs to increase if it is to have any real impact on primary care workload and costs [6-8]. Relatively recent studies from similar interventions such as AskMyGP or eConsult, both part of the United Kingdom’s National Health Service system, have produced mainly qualitative evidence that seems to indicate that their performance meets expectations in terms of access to the health care system but not in terms of patient autonomy [9] and the role of a local champion, or a clinician with a good understanding of the tool and an interest in using it, is key for the intervention to be perceived as useful in the context of routine practice and therefore successful. However, because it is often necessary to combine the service with face-to-face or telephone consultations, it is perceived as an additional administrative burden for doctors [10,11]. To identify the impact of teleconsultation use on professional workload, we must first increase their use [12]. Teleconsultations involving interactions between GPs and hospital specialists have been subject to more extensive investigation and have produced positive results in terms of reducing waiting times and improving coordination with hospitals [13]. They are also well accepted by users [14,15].

### Study Aim

This study aimed to assess the impact of the use of eConsulta on the number of face-to-face visits to PCTs.

## Methods

### Participants

The study was conducted in the Central Catalonia Health Region, a large, mainly rural area that also includes major cities such as Manresa, Igualada, Vic, Solsona, and Berga. The region’s total population exceeds 500,000 people. In this area, 173 GPs had used eConsulta at least once, but most of them had tried it only a few times. The 20 GPs who most frequently used eConsulta and accounted for nearly 70% of the total number of teleconsultations were asked to participate in the study. The invitation was accepted by 18 of the 20 GPs. The study data comes from the administrative data of health provider organizations and covers the period April 8, 2016 to August 18, 2018.

The Territorial Administration Office of the Central Catalonia Region of the Catalan Institute of Health provided the GPs with a register of eConsulta interactions undertaken during the study period. The 18 participating GPs only had access to their own data, for reasons of confidentiality. The text in the subject line and message body was analyzed after the data were anonymized.

### Variables in the Analysis

Each GP recorded three pieces of information for each of their interactions: the type of interaction according to the 13 author-proposed categories (Multimedia Appendix 1); whether they believed a face-to-face visit was avoided, which was defined as the absence of the need for a face-to-face visit following the consultation; and whether they believed the patient would have requested a face-to-face visit had eConsulta not been available. The latter was used as an approximate measure of the possible increased demand resulting from the ease of access to a GP. This subjective information was cross-referenced with information registered by the information systems, which is shown in Table 1. With regard to the ID, it refers to the number of teleconsultations, not patients; therefore, the object of the analysis cannot be inferred as the number of participating patients but the number of interactions. With reference to “Message order,” the interlocutor is inferred based on who initiated the teleconsultations and the order of the messages. Thus, if a teleconsultation is initiated by the patient, it is assumed that the messages that follow alternate between the GP and the patient, although it is possible that either may have written more than one message in succession

**Table 1:** Example of an anonymized administrative record.

ID	Initiated by	Title	Date	Message order	Message
306	Patient	Test message	04/13/2017	1	Good morning XXXXX, it’s an honor to be the first person to use this service. Cheers!
306	Patient	Test message	04/14/2017	2	Good morning XXXXXX, I hope you find the service useful. Goodbye!

The statistical programs Epi Info v.7.2.2.1 (Division of Health Informatics & Surveillance Center for Surveillance, Epidemiology & Laboratory Services, Atlanta, GA), SPSS v.8 (IBM Corp, Armonk, NY) and R v.3.6.1 (R Project) were used for the statistical analyses. The results were considered significant with *P*<.05. The study was approved by the Ethical Committee for Clinical Research at the Foundation University Institute for Primary Health Care Research Jordi Gol i Gurina (registration number P18/023).

## Results

### Descriptive Analysis of the Sample of Teleconsultations

A total of 3559 messages corresponding to 2268 teleconsultations were analyzed (mean 1.57 messages per teleconsultation, SD 0.54). The patient initiated 60.0% (1361/2268) of the teleconsultations, and a significant proportion consisted of a single message that did not generate a response (1040/2268, 45.9%). The remainder consisted of mostly 2-5 interactions (see Table 2). Regarding the format, the messages were composed of a title and text body that were on average 17 and 250 characters long, respectively. Texts of messages written by the GPs were slightly longer than those written by patients (mean 280 vs 190 characters, respectively).

**Table 2:** Frequency of each number of messages per teleconsultation.

Number of messages per teleconsultation	n
1	1040
2	1177
3	40
4	8
5	3

**Figure 1:**
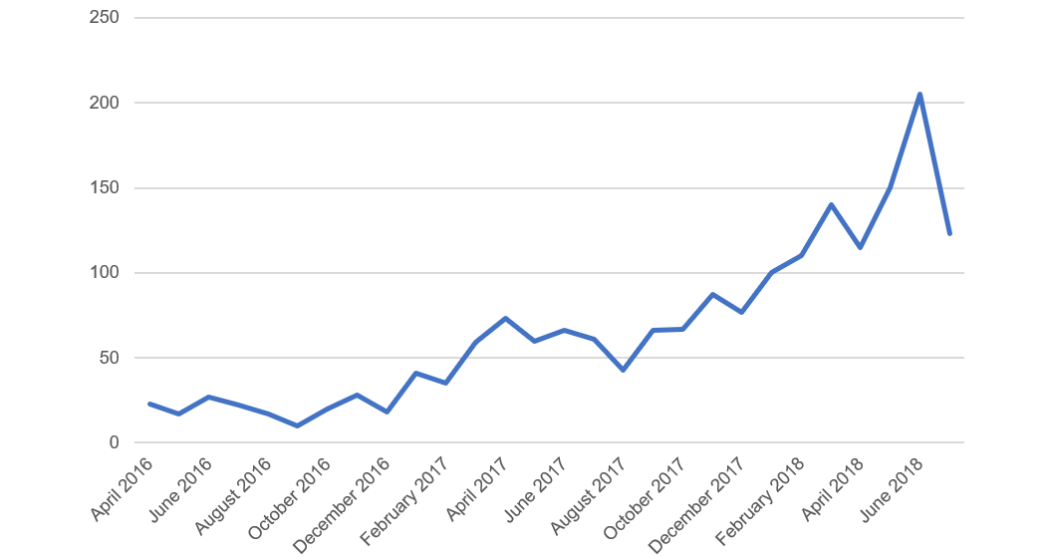
Number of teleconsultations per month during the study period.

The number of teleconsultations fluctuated throughout the year, showing an upward trend in the use of the tool over time, with fluctuations in usage corresponding to the months of greater (winter) and lesser (summer holidays) health care activity (Figure 1).

Most messages were sent to individuals, with one notable exception. A GP sent the same message to multiple patients simultaneously, with the following text: “The anti-flu campaign is about to begin. It is recommended that anyone aged over 60 and those suffering from a respiratory infection, heart or kidney disease or diabetes ought to be vaccinated from 23-10-17. See attached file. Ask your nurse for an appointment. Sincerely, Dr XXXX”.

### Types of Teleconsultations

The information in Table 1 was supplemented by linking each case with an anonymous ID and the collection of data from the GPs in the form of a template that included three questions. In response to the question “Did eConsulta avoid a face-to-face visit?”, the GP replied in the affirmative for 88% of the cases. In terms of the types of enquiries, the most common were related to the management of test results, clinical enquiries, and repeat prescriptions, while the least frequent were requests for clinical reports and sick notes and queries related to vaccinations and the use of anticoagulants. Because 112 conversations were not classified correctly, they were excluded from the analysis, leaving 2156 conversations (see Table 3). Errors and test messages constituted some 7% of the total, which demonstrates the experimental nature of the intervention. The subsequent analysis excludes messages corresponding to categories 11 (messages sent in error), 12 (other), and 13 (test messages).

**Figure 2:**
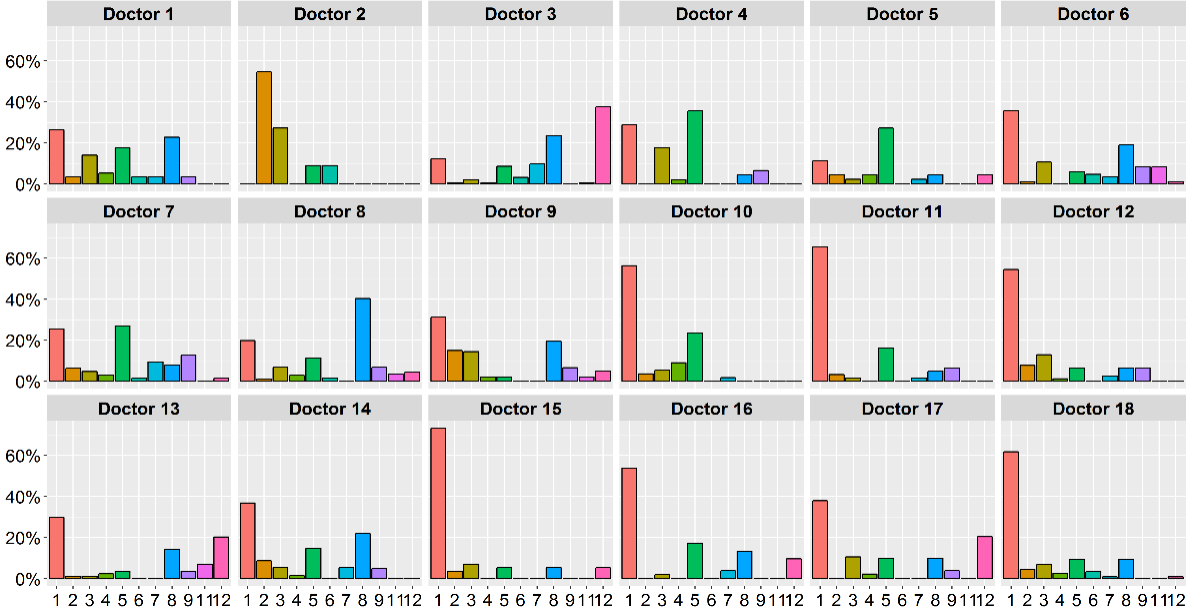
Type of teleconsultation by professional.

Finally, in terms of the type of teleconsultations engaged in by each GP (Figure 2), while Type 1 was the most frequent, the GPs (numbered 1 to 18 in the figures) favored a specific purpose. For example, see the use by GPs 2, 5, and 8 in Figure 2.

**Table 3:** Number of teleconsultations, by type.

Type of consultation	n (%)
1. Management of test results	758 (35.2)
2. Temporary disability management	113 (5.2)
3. Arranging an appointment	160 (7.4)
4. Requesting a clinical report/sick note	37 (1.7)
5. Repeat prescription	262 (12.2)
6. Vaccinations	21 (0.97)
7. Other administrative issues	67 (3.1)
8. Medical enquiries	345 (16.0)
9. Issues regarding medicines	79 (3.7)
10. Queries regarding anticoagulants	1 (0.0)
11. Messages sent in error	45 (2.1)
12. Other	144 (6.7)
13 Test messages	124 (5.8)

### Does eConsulta Reduce the Number of Face-to-face Visits?

**Figure 3:**
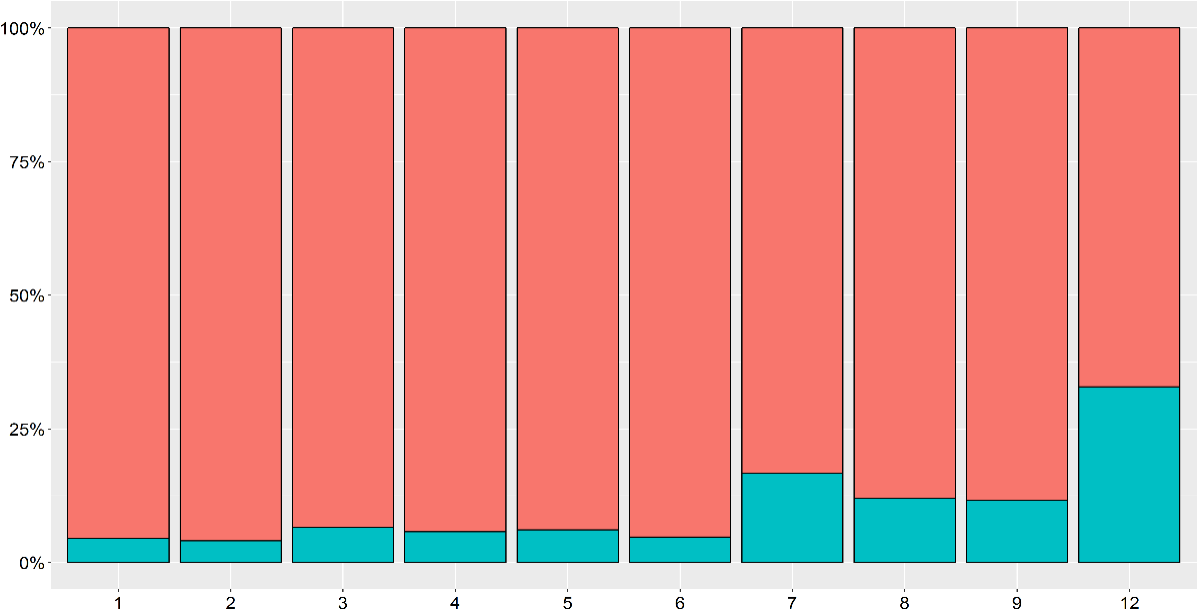
Results of whether each type of teleconsultation avoided a face-to-face visit (upper bar=Yes). Refer to Table 3 for expansions of numbers used on the x-axis.

In response to the question “Has the online consultation avoided a face-to-face visit?”, GPs answered yes for 87.9% (1918/2180) of the teleconsultations. A breakdown of the results (Figure 3) shows that the ability to decrease the number of face-to-face visits (mean 0.89, SD 0.08) is largely uniform in terms of the type of consultation. The teleconsultations around which the GPs were the least decisive corresponded to the “Other” category, although there was no indication as to why they fail to avoid a face-to-face visit.

**Figure 4:**
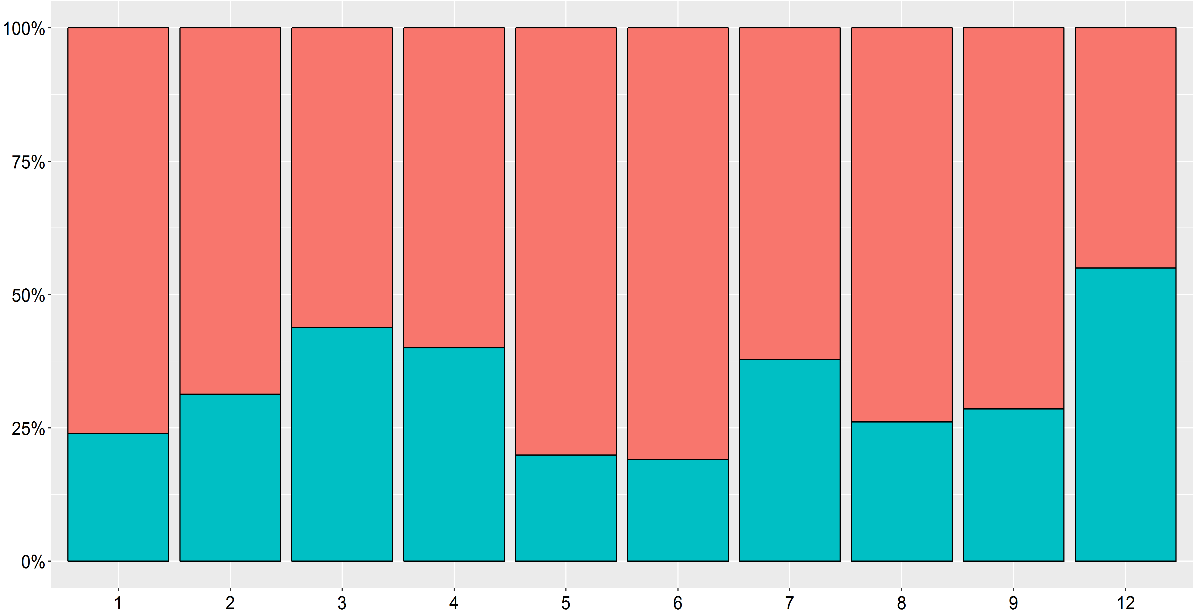
If eConsulta was not available, whether the patient would have visited the general practitioner’s surgery (upper bar=yes), by type of visit. Refer to Table 3 for expansions of numbers used on the x-axis.

In response to the question “In the absence of a service like eConsulta, would the patient have made a face-to-face visit?”, GPs answered yes for 72.2% (1574/2180) of the teleconsultations, suggesting that the ease of contact with the GP reduced demand for 27.7% (604/2180) of the cases. In the analysis by type of consultation (Figure 4), tool-facilitated ease of contact occurred mainly for type 12 consultations (Other: mean 0.67, SD 0.12). Therefore, based on the equation (88% x [1-28%]), telemedicine could replace 63%-88% of conventional appointments, which supports the findings of other studies [11].

### Other Analyzed Relationships

Because teleconsultations consisting of a single message were so frequent, we performed additional analyses with these data. They were related primarily to the “Management of test results” category. These correspond to GPs who provide test results that do not require a specific comment (Multimedia Appendix 2). These tests are known as “complementary” in the clinical setting, since they complement the clinical assessment and it makes sense to analyze them outside the GP office as they are normal and of no added clinical value.

Finally, the relationships between the ability to resolve an issue, who initiated the interaction, and the message length were studied. First, who initiated the teleconsultation did not determine its degree of resolution, since the frequency of resolution by teleconsultations was not statistically different between consultations initiated by the patient or GP (*P*=.045; Table 4). With regard to message length, we analyzed whether longer conversations are better able to resolve issues. Although the message length might serve as an indicator of the degree of complexity of the problem, very short messages may not be sufficiently descriptive to resolve the query. The messages were divided equally into three length categories: short, medium, and long. Message length had a statistically significant impact, with longer messages increasing the likelihood of the consultation being satisfactorily concluded (odds ratio 1.66, 95% CI 1.32-2.07; *P*<.001; Multimedia Appendix 3).

**Table 4:** Degree of resolution in avoiding face-to-face visits, by initiator.

Initiator of the teleconsultation	The teleconsultation avoided a face-to-face visit	%
General practitioner	No	11.87
	Yes	88.12
Patient	No	12.56
	Yes	87.43

## Discussion

This study offers useful pointers for policy making since it suggests that eConsulta is a tool that can reduce the number of face-to-face consultations in a primary care setting. The study also provides information about the visit types for which eConsulta is most likely to be used by both patients and GPs. Although a recent study suggested that the topics suitable for teleconsultations will vary depending on the clinical settings and on the individuals who use the tool [16], the current study found that eConsulta is mainly used for the management of test results, to resolve clinical problems, and for queries related to repeat prescriptions. It is worth noting that the second most frequent reason for sending a message was for a medical enquiry, demonstrating that there is demand for a non-face-to-face means to resolve health issues.

This study has several limitations. First, despite efforts to systematize the approach, the evaluation was purely subjective. However, despite the lack of a quantitative approach, it seems logical that GPs, in the absence of a conflict of interest, can realistically classify the intervention’s ability to avoid a face-to-face visit. Second, eConsulta is still in the early stages, and GPs are still experimenting with the service, as demonstrated by the high proportion of test messages, message errors, and messages that failed to receive a response. This analysis represents the first steps in the use of the tool. Third, using evaluations by GPs who use the tool most introduces significant bias into the evaluation, since they may not be representative of the population. Future studies ought to assess the impact of the intervention on objective measures.

Although it is challenging to incorporate teleconsultations into the daily clinical workflow, as reported by other authors [17,18], the bottleneck in the deployment of the intervention may not originate with the GPs but instead with the patients. Few patients make use of their Personal Health Folder, the portal through which the eConsulta service is available. As a result, it would be worthwhile to investigate the relationship between face-to-face visits and the use of ICT tools in a more general sense. Future research should include the role of telephone consultations, another type of non-presential service available to GPs, to assess the impact of one type of consultation on the other.

## Appendix

Multimedia Appendix 1 Reasons that patients and general practitioners use eConsulta.

Multimedia Appendix 2 Teleconsultations that consisted of a single message, by type.

Multimedia Appendix 3 According to message length, whether the patient would have visited the general practitioner’s office if eConsulta had not been available (left bar=Yes).

## Abbreviations

GP: general practitioner.
ICT: Information and Communication Technologies.
PCT: Primary Care Team.
