# Supplementary material for: General Practitioners’ Perceptions of Whether Teleconsultations Reduce the Number of Face-to-face Visits in the Catalan Public Primary Care System: Retrospective Cross-Sectional Study
Source: J Med Internet Res. 2020 Mar 16;22(3):e14478. doi: 10.2196/14478 (PMC7105927; doi:10.2196/14478)
Supplement: Multimedia Appendix 1 [file jmir_v22i3e14478_app1.docx]

ADMINISTRATIVE

**1.** **Management of test results**

o The patient provides the results of tests from an external center so they are recorded in their medical history.

o The GP provides normal test results.

o The GP deals with test-related questions from the patient.

o The GP requests tests after conducting a follow-up teleconsultation.

**2.** **Temporary disability management**

o The patient communicates changes to their health related to an upcoming temporary disability.

o The GP tracks the progress of a temporary disability in conjunction with face-to-face visits.

**3. Management of visits/referrals**

o The patient has an enquiry which the GP thinks ought to be dealt with by a specialist and refers them. They can also report incidents resulting from any referrals made.

o The GP resolves incidents relating to the timing of visits.

o The GP cancels visits from other clinicians in cases in which the problem has been resolved following completion of the econsultation.

o Validation of appointments with other specialists where the citizen needs more information about the motivation of the appointment.

**4. Request for a clinical report/sick note**

o The patient asks for a report/sick note while consulting their medical history.

o The GP asks the patient for more information in order to prepare the report.

**5. Repeat prescriptions**

o The patient asks for their prescription to be updated if it has been modified by an external specialist, either because they do not use it or because it has expired.

o The GP warns the patient that their prescription is about to expire and updates it.

o The GP cancels an unnecessary prescription following an econsultation.

**6.** **Vaccinations**

o Updates of immunization schedules and general enquiries regarding vaccinations

o Questions concerning vaccinations for travel overseas

**7. Other administrative issues**

- Any administrative procedure that can be resolved without being physically present

MEDICAL

**8. Medical enquiries**

- The patient has a question about their health that can be resolved without a physical examination. They can also attach photographs to accompany the description.

**9. Issues regarding medicines**

- The patient asks a question about a prescription.

**10. Questions regarding anticoagulants and dosage**

OTHER

**11.** **Messages sent in error: the patient made a mistake**

**12. Other**

**13. Test messages**
