# Supplementary figures and images for: General Practitioners’ Perceptions of Whether Teleconsultations Reduce the Number of Face-to-face Visits in the Catalan Public Primary Care System: Retrospective Cross-Sectional Study
Source: J Med Internet Res. 2020 Mar 16;22(3):e14478. doi: 10.2196/14478 (PMC7105927; doi:10.2196/14478)

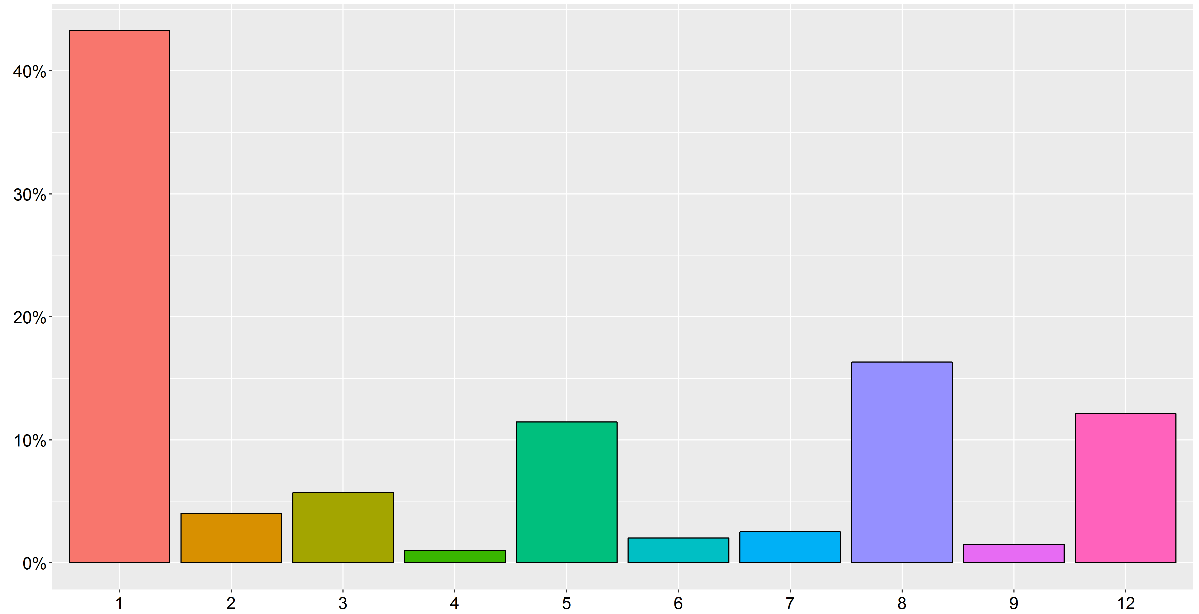

Supplement: Multimedia Appendix 2 [file jmir_v22i3e14478_app2.png]

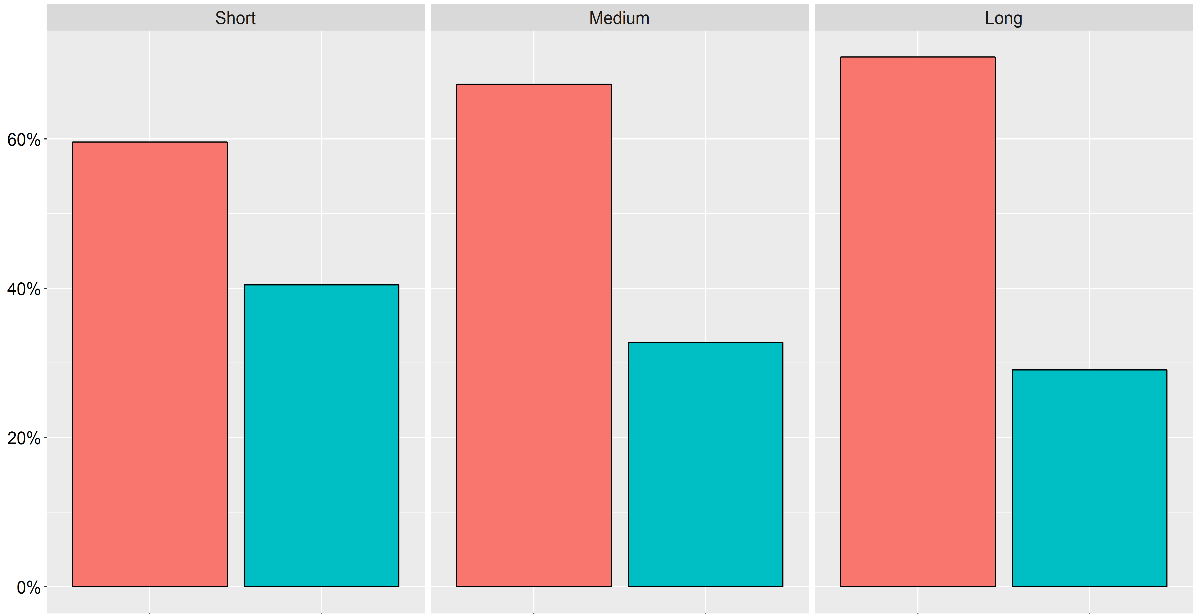

Supplement: Multimedia Appendix 3 [file jmir_v22i3e14478_app3.png]
